# Influence of aging on dermal elastin fiber architecture and skin firmness assessed by finite element modeling

**DOI:** 10.1038/s41598-025-14393-2

**Published:** 2025-08-05

**Authors:** Fei Jiang, Takeshi Tohgasaki, Mayuko Kami, Ryota Sanuki, Yuya Nakata, Shinya Kondo, Xian Chen

**Affiliations:** 1 Department of Mechanical Engineering, Graduate School of Sciences and Technology for Innovation, Yamaguchi University, Ube, Yamaguchi 755-8611 Japan; 2 FANCL Research Institute, FANCL Corporation, Yokohama, Kanagawa 244-0806 Japan

**Keywords:** Elastin fiber, Skin firmness, Finite element modeling, Computational biophysics, Tissues, Biomedical engineering, Computational science

## Abstract

Skin firmness and elasticity are largely determined by the dermal extracellular matrix, particularly the elastin fiber network. Age-related degradation of elastin alters its architecture, contributing to diminished skin resilience. However, the quantitative relationship between elastin fiber geometry and macroscopic skin firmness remains incompletely understood. In this study, we developed a novel computational framework integrating realistic 3D elastin fiber geometries–extracted from confocal microscopy images of human abdominal skin samples (Caucasian females, aged 38–78 years)–into a finite element (FE) model of the dermal matrix. The elastin networks were explicitly represented as beam elements within the FE domain. Unconfined compression simulations were conducted to evaluate skin’s elastic resistance force and correlate it with quantified geometric parameters of the elastin networks. The results revealed a significant age-dependent decline in skin firmness, strongly associated with reductions in fiber diameter, fiber count, volume fraction, network connectivity (as indicated by increased fragmentation and reduced maximum cluster size), and the proportion of vertically oriented fibers. Among these, fiber count and maximum cluster size were the most important predictors of skin firmness. This study provides quantitative, mechanistic insights into how specific architectural alterations in elastin fibers directly impact the mechanical properties of aging skin. These findings emphasize the critical role of elastin network integrity and structural organization in maintaining skin function and offer a compelling rationale for therapeutic or cosmetic strategies aimed at preserving or restoring the elastin framework to maintain skin firmness.

## Introduction

Human skin, the largest organ of the body, serves as a crucial interface between the internal environment and the external world. Its complex structure is broadly categorized into three distinct layers: the epidermis, the outermost protective layer; the dermis, the underlying connective tissue layer providing mechanical strength and elasticity; and the hypodermis or subcutaneous tissue, which primarily functions in insulation and energy storage. Among these layers, the dermis is recognized as the principal load-bearing component, largely responsible for the skin’s overall mechanical behavior^1^. This layer is a rich composite of various structural and functional elements, including collagen fibers, elastin fibers, and the surrounding amorphous ground substance^2^. Understanding the mechanical behavior of the dermis is of paramount importance in a diverse range of applications. In dermatology, it aids in diagnosing skin conditions and evaluating the effectiveness of treatments^3^. The cosmetic industry relies on this knowledge to develop products that target skin firmness and elasticity and reduce the signs of aging^3^. In surgery, understanding skin mechanics is critical for planning incisions, designing skin flaps, and optimizing wound closure^1^. Therefore, the mechanical properties of the dermis are intrinsically linked to skin health, susceptibility to disease, the process of aging, and the efficacy of various therapeutic interventions^4^.

Within the dermis, elastin fibers are particularly crucial for skin firmness and elasticity, enabling skin recoil after deformation, especially under low strain^5,6^. Skin firmness refers to the skin’s resistance to deformation under load, whereas skin elasticity describes its ability to return to its original shape after deformation. Elastin contributes significantly to the initial low stiffness observed in the skin’s mechanical response before stiffer collagen fibers engage, possessing much greater stretchability than collagen^7^. These elastic fibers are organized into an intricate, three-dimensional network within the dermis, with fibers oriented both perpendicularly and parallel to the skin surface. This arrangement allows the skin to accommodate movement and stretching from various directions^8^. As the skin ages, the elastin fibers undergo a process of degradation influenced by several mechanisms^9^. Enzymatic action plays a role, with enzymes such as elastases and matrix metalloproteinases (MMPs) breaking down elastin. Oxidative damage, caused by reactive oxygen species, also contributes to elastin degradation^9^. Glycation, the non-enzymatic reaction of sugars with proteins leading to the formation of advanced glycation end products (AGEs), further compromises the integrity of elastin fibers^9^. Ayadh et al.^10–12^ conducted in vivo experiments to measure the fluorescence intensity of glycation products and to assess the influence of fiber organization on the mechanical properties of skin. Additionally, the repeated mechanical stretching and relaxation of the skin over a lifetime can lead to mechanical fatigue of the elastin fibers^9^. This cumulative degradation results in a reduction in the amount of functional elastin present in the dermis and a disorganization of the normally intricate elastic fiber network leading to a loss of skin firmness and the formation of wrinkles^13,14^. Kondo et al.^15^ found that elastic fiber deterioration worsened after 40 years of age, correlated with reduced skin recovery and increased wrinkles. Such degradation of elastin fibers leads to a significant change of its geometric characteristics during aging, which highly related to the mechanical behavior of the dermis. Studies have shown that elastin fibers become noticeably shorter with age, while their thickness increases, resulting in more compact fibers compared to the elongated structures observed in youthful skin^9,16^. Three-dimensional analysis further confirms that aged skin, particularly in areas like the eyelids, exhibits consistently shorter fibers. In addition to dimensional changes, elastin fibers undergo pronounced alterations in curvature, shifting from a relatively straight configuration in young skin to increasingly curved and undulating paths with age^17^. In severely aged skin, fibers may even adopt a spherical appearance, disrupting the mechanical integrity of the network and contributing to skin laxity. Moreover, the spatial arrangement of elastin fibers progressively deteriorates with age, transitioning from an initially well-organized circumferential or perpendicular orientation to a more irregular and heterogeneous distribution, especially within the deeper layers of the dermis^17,18^. The reduction in fiber branching and the progressive fragmentation of the elastin network further weaken its structural integrity. However, the quantitative correlations between the changes in elastin fiber morphology and organization and skin firmness still need to be further clarified.

To describe the skin mechanics, most of the works are focusing on the developing of the constitutive model of the skin^19,20^. Phenomenological models of nonlinear elasticity are frequently employed in the literatures to characterize the mechanical behavior of skin^1,14^. However, their applicability is constrained by several limitations, including a lack of direct physical interpretability of the obtained parameters^20^. The constitutive models employed in these studies were derived from foundational frameworks such as the Ogden model^21^, the Mooney-Rivlin model^22,23^, and the Fung model^24^. Subsequently, semi-structural models incorporating distributed fibers were introduced, representing the fibrous network of biological tissues as fiber families with varying orientations embedded within an isotropic matrix^25–28^. Most of these models treat elastin fibers as a continuous reinforcing phase dispersed within the ground substance of the dermis. In these models, tissue anisotropy is primarily attributed to the dispersion of fibers. This distribution is characterized either by a finite set of preferred directions or by accounting for fiber dispersion around the principal orientations. Groves et al.^25^ accounted for fiber contributions by introducing a transversely isotropic hyperelastic constitutive model incorporating three distinct fiber families. This framework effectively captures the anisotropic mechanical behavior of skin, which arises from the orientation and distribution of collagen and elastin fibers within the tissue. The model assumes a layered fiber arrangement parallel to the outer surface of the dermis, making it transversely isotropic only at the level of an individual layer. However, it does not account for potential interactions among the three fiber families, which are generally non-orthogonal. Additionally, the exclusion of an out-of-plane fiber component contradicts multiple experimental findings. Limbert^26^ proposed a decoupled invariant formulation that allows for the representation of the tissue’s response to mechanical loads, taking into account the orientation and distribution of fibers within the tissue matrix. This approach enables the model to reflect the complex interactions between the fibers and the surrounding matrix. The Limbert model^26^ initially demands the identification of 23 physical parameters. While the parameters related to fiber properties can be implicitly inferred from the detailed structural information about the skin at the microscopic level obtained through imaging techniques, the substantial number of variables involved does not ensure a unique solution when optimizing model fit with experimental data. Later, Pond et al.^27^ have further developed this model to link intrinsic aging to microstructural parameters in this constitutive models that account for changes in elastin and collagen networks. Their simulation results suggested that degradation of the elastin meshwork and variations in the anisotropy of the collagen network contribute to macroscopic tissue stiffening observed with aging. Guissouma et al.^28^ adopted a similar idea to develop a multiscale four-layer FE model to predict the effects of collagen fibers on skin behavior under tension, considering the nonlinear elasticity of collagen fiber density, radius, undulation, and orientation. Despite their advantages, semi-structural models often oversimplify the actual fiber arrangements within the dermis. To date, no study has explicitly incorporated the real geometries of elastin fibers into skin modeling.

To overcome the limitations of these idealized or semi-structural representations, the field of biomechanics has increasingly moved towards image-based finite element modeling. In this paradigm, high-resolution scans from modalities like MRI or CT are used to generate subject-specific meshes that faithfully replicate anatomical geometry, while microscopic imaging (e.g. confocal microscopy) can capture fine-scale tissue architecture for micro-level models^29–32^. By explicitly meshing these real geometries, these models can provide unprecedented insight into how microstructure dictates tissue-level mechanical behavior, capturing complexities that homogenized models cannot. Studies have shown that incorporating actual tissue morphology (e.g. layered microstructures of skin or collagenous networks) into FE models can significantly influence predicted mechanical behavior compared to idealized geometries^33^. However, despite the power of this approach, its application to explicitly model the 3D geometry of the dermal elastin network for the purpose of studying skin aging remains largely unexplored.

This research aims to integrate the precise geometry of elastin fibers into a computational model to clarify the relationship between fiber structure and skin firmness using the FE method. In this model, elastin fibers are represented as three dimensional beam elements embedded within a continuum-based dermal matrix. A key advantage of this approach is that the fiber geometries are directly extracted from 3D imaging data of actual skin and explicitly incorporated, allowing for a realistic representation of elastin’s contribution to overall mechanical properties. This methodology enables an in-depth analysis of how fiber characteristics–such as length, diameter, and orientation–affect the skin’s mechanical response. Additionally, the model can incorporate fiber orientation distribution information to account for the non-uniform alignment of elastin fibers. By identifying the key structural factors contributing to age-related firmness loss, this study provides a quantitative framework for understanding the biomechanical role of elastin fiber geometry. These insights may support the development of anti-aging treatments, cosmetic applications, and biomedical engineering strategies aimed at preserving or restoring skin firmness.

## Materials and methods

### 3D Imaging and data acquisition

**Fig. 1:**
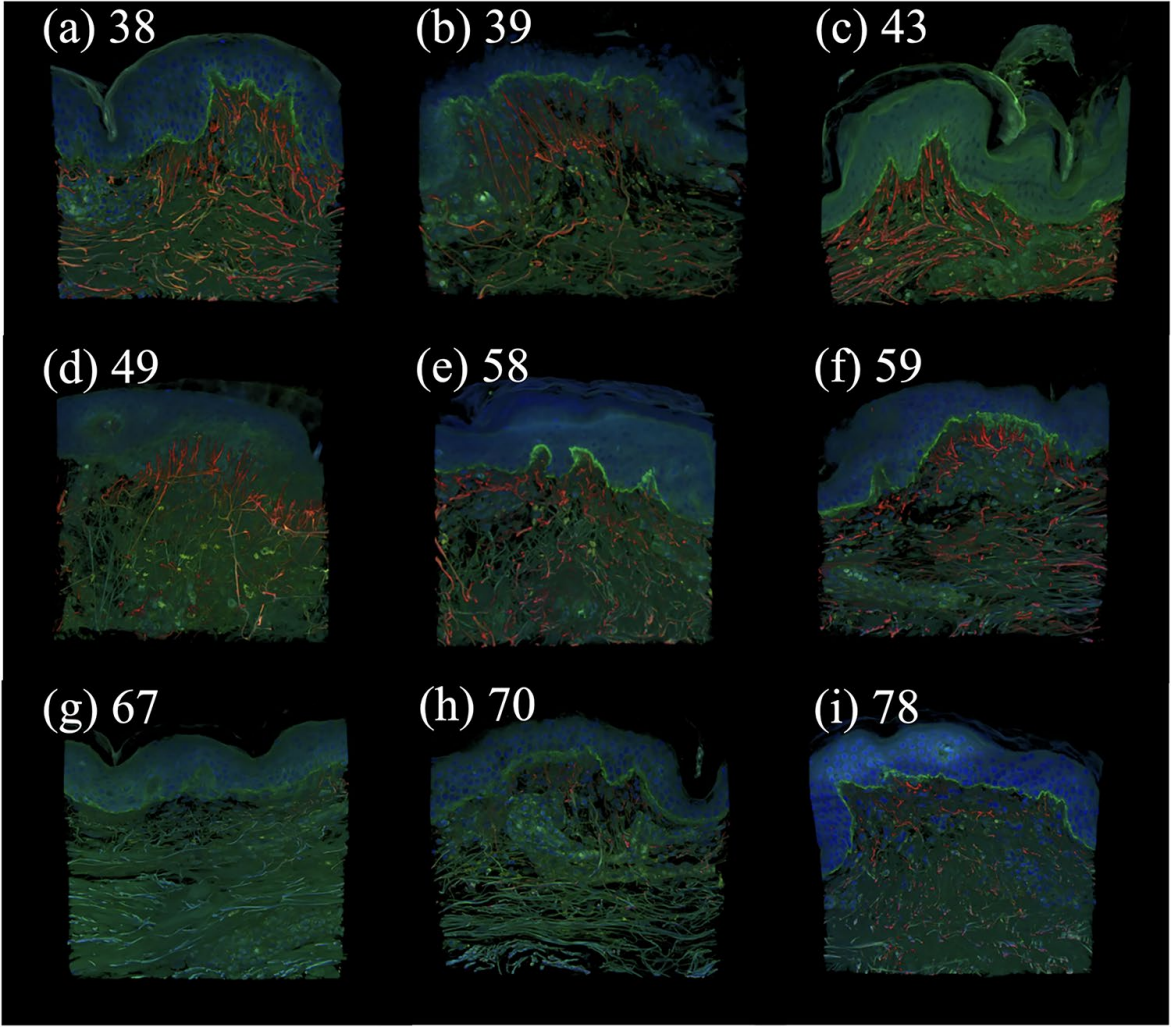
3D images of human skin at different ages labeled with the corresponding ages of the individuals: (**a**) 38, (**b**) 39, (**c**) 43, (**d**) 49, (**e**) 58, (**f**) 59, (**g**) 67, (**h**) 70, and (**i**) 78 years old, in which red represents elastin fibers, green represents collagen VII, and blue represents cell nuclei.

Human abdominal skin tissues were procured from Biopredic International (Rennes, France) through the Japanese import agent KAC Co., Ltd. (Kyoto, Japan). The study cohort consisted of nine Caucasian female donors with the following demographic details: 38 years (BMI: 28), 39 years (BMI: 30), 43 years (BMI: 30), 49 years (BMI: 33), 58 years (BMI: 28), 59 years (BMI: 29), 67 years (BMI: 26), 70 years (BMI: 26), and 78 years (BMI: 35). Those skin samples were procured from living donors undergoing cosmetic surgery (abdominoplasty). Immediately following excision, all tissue samples were fixed in 4% paraformaldehyde (PFA) in phosphate-buffered saline (PBS) and transported to our laboratory in Japan while submerged in the same fixative solution. It is important to note that the process of excising tissue from the body releases the physiological in vivo residual tension. As the samples were chemically fixed immediately after this process, the initial state of the tissue for both imaging and subsequent computational modeling was considered to be a relaxed, stress-free reference configuration. Biopredic International, an ethically compliant supplier, acquired all tissues with proper informed consent from donors, in accordance with their institutional policies and ethical guidelines. The procedures for staining and 3D image acquisition were performed according to the methods detailed by Tohgasaki et al.^34^. Each fixed skin tissue was sliced into 1-mm thick sections in an epidermis–dermis orientation. For immunostaining, skin tissues were first treated with $\hbox {StartingBlock}^{\textrm{TM}}$ Blocking Buffer (Thermo Fisher Scientific; Waltham, MA, USA) to block non-specific antibody binding. The primary antibodies–anti-tropoelastin mouse monoclonal antibody (Santa Cruz Biotechnology; Dallas, TX, USA) and anti-collagen VII rabbit polyclonal antibody (Abcam; Cambridge, UK)–were diluted to 1:200 in the blocking buffer and incubated with the tissues for two nights at $4^\circ$C. Subsequently, tissues were treated with the corresponding secondary antibodies–anti-mouse IgG (H+L) Alexa Fluor®488 (Thermo Fisher Scientific) and goat anti-rabbit IgG (H+L) Alexa Fluor®647 (Thermo Fisher Scientific)–diluted to 1:1000 in the blocking buffer. Nuclei were visualized using 4’,6-diamidino-2-phenylindole (DAPI; DOJINDO Laboratories, Kumamoto, Japan) at a 1:5000 dilution. The secondary antibodies and DAPI were incubated simultaneously for one night at $4^\circ$C. Post-staining, tissues were rendered transparent using Rapiclear 1.49 (SunJin Lab Co.; Hsinchu, Taiwan). 3D images depicting nuclei, tropoelastin, and the basement membrane constituent collagen VII were captured using an A1R confocal laser scanning microscope (Nikon, Tokyo, Japan) equipped with a 60x oil immersion objective lens. The final 3D reconstructions of human skin from donors aged 38 to 78 years are shown in Fig. 1, subfigures (a) through (i), respectively, highlighting age-related structural alterations in dermal components over time.

### FE model reconstruction and simulation setup

**Fig. 2:**
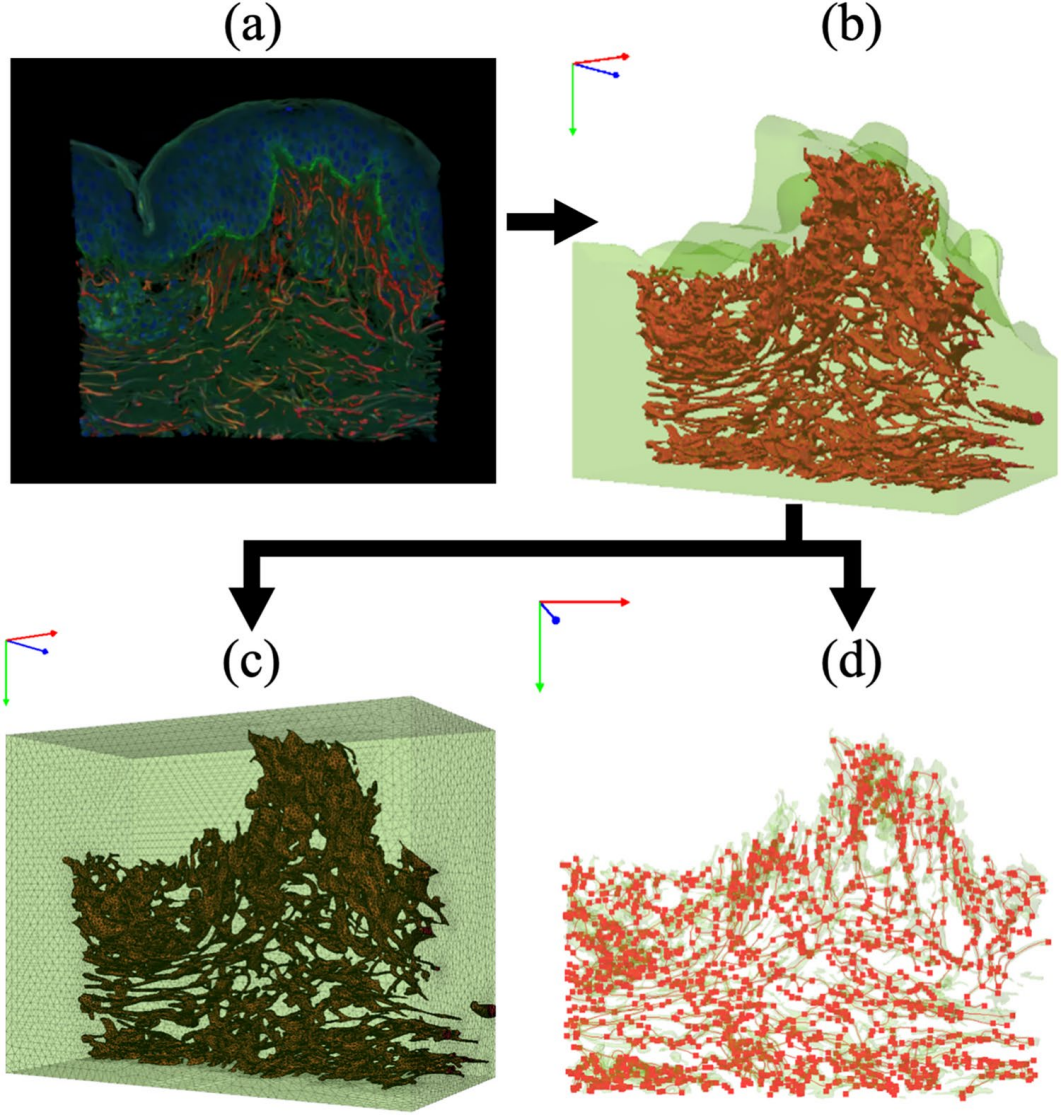
Workflow for constructing a computational model of the dermis incorporating real elastin fiber geometry. (**a**) 3D reconstructed image of human dermis showing elastin fibers (red) extracted from confocal microscopy data. (**b**) Segmented and smoothed elastin fiber network geometry obtained from the image data. (**c**) Embedding of the reconstructed elastin fiber geometry into a 3D FE mesh of the surrounding dermal matrix. (**d**) Representation of elastin fibers as beam elements for FE analysis, capturing their individual geometries and orientations within the dermis.

**Fig. 3:**
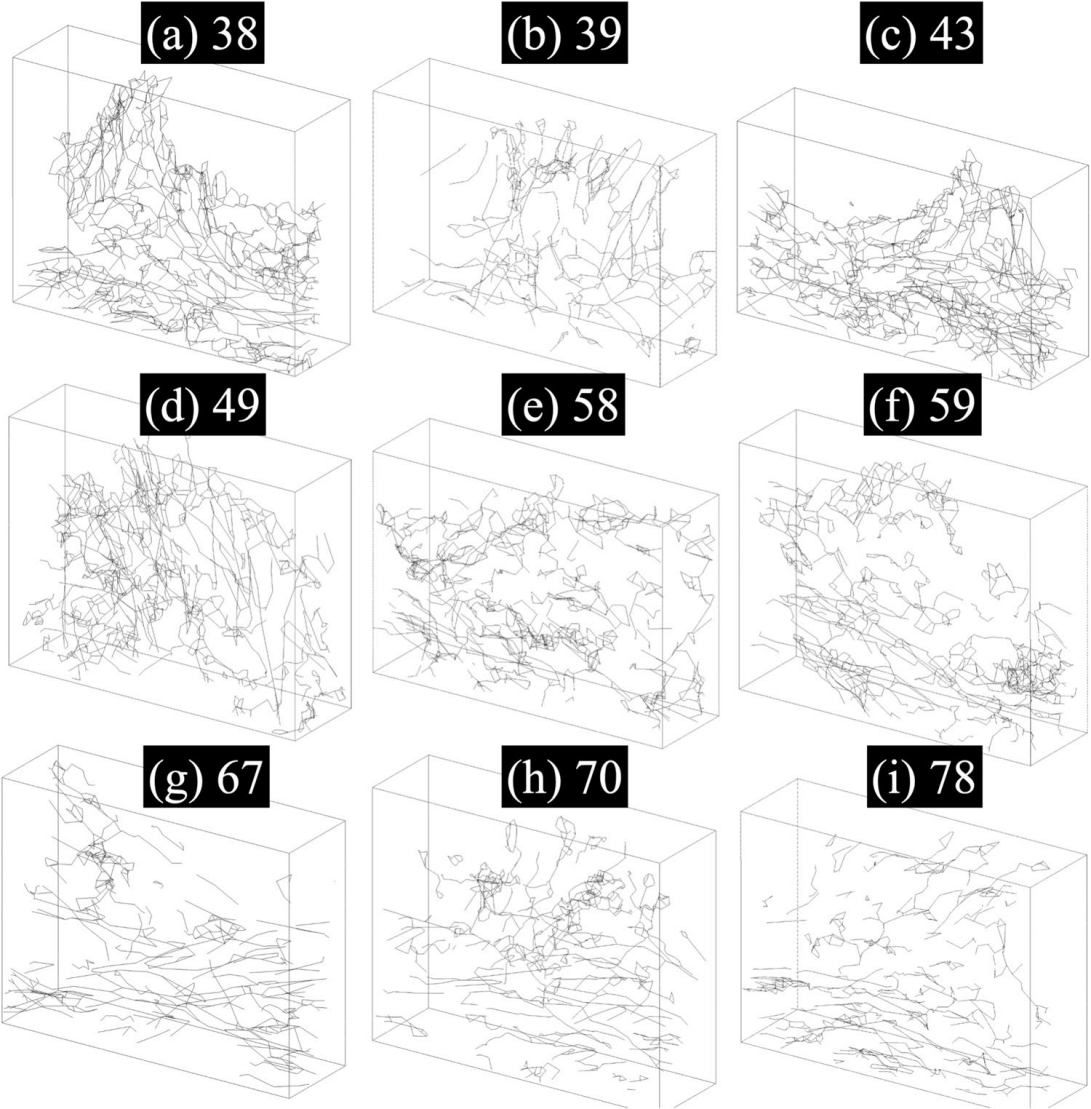
Age-dependent changes in the three-dimensional geometry of elastin fibers in human dermal tissue. Panels (**a–i**) show reconstructed elastin fiber networks from skin samples of individuals aged 38 to 78 years. The fibers are visualized as centerlines extracted from segmented 3D microscopy images. Younger samples (e.g., 38, 43 years) exhibit dense, vertically aligned, and highly interconnected networks, while older samples (e.g., 67, 70, 78 years) display sparse, fragmented, and predominantly horizontal fiber orientations. The progressive degradation in fiber architecture with age reflects structural remodeling of the dermal matrix, which may contribute to the observed decline in skin firmness and elasticity.

To construct a computational model that accurately incorporates the real geometry of elastin fibers within the dermis, a multi-step image-based modeling workflow was employed (Fig. 2). First, confocal microscopy was used to acquire high-resolution 3D image stacks of human dermal tissue, in which elastin fibers were visualized using the method described in the previous section (Fig. 2a). These volumetric images were then processed to segment and isolate the elastin fiber structures using advanced image analysis techniques, such as thresholding and morphological filtering, enabling the extraction of continuous fiber geometries (Fig. 2b). To reduce noise, we applied a 3D median filter with a 3x3x3 voxel kernel to the raw image stacks. For segmentation, we utilized a single-intensity thresholding method, with threshold values manually determined for each 3D image stack. The value was chosen interactively to ensure it optimally captured the brightly stained elastin fibers while minimizing the inclusion of background noise, as confirmed by visual inspection of the resulting segmentation. This approach allowed for careful adjustment to account for slight variations in staining intensity between samples. The resulting binary segmentation was then refined using 3D morphological operations. A morphological opening (an erosion followed by a dilation) with a 3x3x3 voxel spherical structuring element was used to remove small, isolated noise particles. This was followed by a morphological closing (a dilation followed by an erosion) with the same structuring element to fill small gaps within the fibers, ensuring their continuity for structural analysis. Finally, a Laplacian smoothing algorithm was applied to the surface of the segmented binary volume. This step served to reduce voxel-based “stair-step” artifacts, creating a smoother and more realistic fiber geometry. The dermal matrix was meshed using 4-node linear tetrahedral elements (Fig. 2c). A detailed breakdown of the number of elements for each individual model is provided in the Supplementary Information (Supplementary Table S1). All image processing tasks and the generation of the tetrahedral mesh were performed within the commercial software package Simpleware ScanIP (v2023.03, Synopsys). To capture the mechanical contributions of elastin fibers, each segmented fiber was modeled as a 2-node, 3D Euler-Bernoulli beam element and embedded within the tetrahedral mesh representing the isotropic dermis domain (Fig. 2d). This approach was adopted because the load-bearing characteristics of elastin fibers–notably their flexibility, extensibility, and bending resistance–are analogous to those of beam structures. The nodal points of the beam elements were defined based on the centerline coordinates of the segmented fibers. To integrate the beam elements with the tetrahedral mesh of the dermal matrix, the nodal positions of the beam elements were slightly adjusted and then merged with the nearest nodes of the dermal matrix’s tetrahedral mesh. This modeling strategy enables the investigation of the mechanical behavior of the dermis with explicit consideration of the spatial distribution, orientation, and morphology of the elastin network, offering a significant advancement over traditional phenomenological or homogenized representations. Using three dimensional beam elements instead of continuum elements to represent fibers enhances computational efficiency while preserving essential mechanical characteristics. One limitation of this method is that it inherently neglects transverse deformations of fibers and the shear interaction between the fiber and matrix. The thin epidermis is ignored in our numerical model. This simplification is justified because the much thicker dermis is the principal load-bearing component responsible for the skin’s bulk elasticity, and our study’s primary focus is on the role of dermal elastin fibers. To mitigate the effects of surface irregularities in the individual skin samples, all FE models were standardized to a rectangular geometry with the same section area ($343.57\times 99.75 \mu m ^2$). This approach also facilitated the subsequent application of boundary conditions to the top surface. The height of each model was determined by the depth of its respective 3D confocal image stack. The specific final dimensions for each of the nine models are provided in the Supplementary Information (Supplementary Table S2). The reconstructed 3D distributions of beam elements representing dermal elastin fibers across all examined age groups are shown in Fig. 3, subfigures (a) to (h), respectively.

**Fig. 4:**
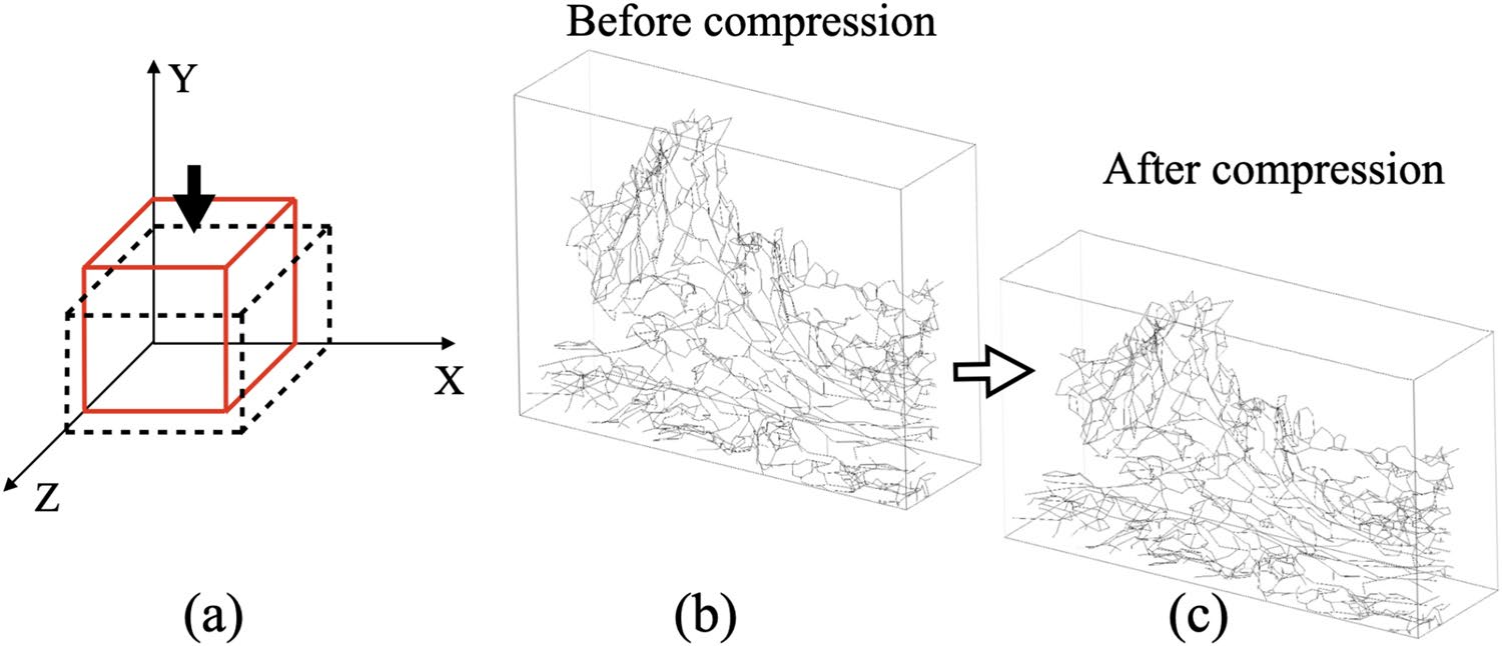
The unconfined compression simulation setup for the skin model. (**a**) Boundary conditions: arrows show directions of applied displacements on the top surface; the left surface is constrained in the horizontal direction, the bottom surface is fixed in the vertical direction, and the back surface is restricted in the depth direction; Multi-Point Constraint (MPC) conditions are imposed on the other surfaces to maintain their planar shape during deformation (**b**) Elastin fiber network before compression; (**c**) Elastin fiber configuration after compression.

This study utilized a quasi-static unconfined compression (UCC) simulation to investigate the mechanical response of skin tissue under deformation. The boundary conditions (Fig. 4a) were defined as follows: the left surface of the model was constrained horizontally (x-direction), the bottom surface was fixed vertically (y-direction), and the back surface was restricted in the depth direction (z-direction). A prescribed vertical displacement was applied to the top surface, compressing the model to 80% of its original height. This 20% compression level was chosen as it is substantial enough to elicit a clear mechanical response from the elastin network across all samples, while also representing a physiologically plausible deformation that avoids numerical instabilities associated with excessive element distortion in the simulation. Multi-Point Constraint (MPC) conditions were implemented to ensure the front and right surfaces maintained planarity. To account for large deformations, the dermal matrix was modeled using the Saint Venant–Kirchhoff hyperelastic formulation, incorporating near-incompressibility to accurately represent its mechanical behavior. This model was chosen intentionally to simplify the system and focus the analysis squarely on the primary variable of interest: the 3D geometry of the elastin fiber network. By using a simpler, two-parameter (Lamé parameters) model for the matrix, we minimized the number of free parameters and ensured that the differences observed between our simulations were attributable to the measured architectural changes rather than the fitting of a more complex material model. The beam elements representing the elastin fibers were separately modeled with directional properties and embedded to capture anisotropic effects. Each beam element was modeled with a circular cross-section, with diameters assigned based on measurements from segmented image data. The elastic moduli were set to 0.5 MPa for elastin fibers^35^ and 80 kPa for the dermal matrix^1^. The Poisson’s ratio is set to 0.48. The commercial finite element solver MSC Marc (v2023, Hexagon) was used to define the material properties, apply boundary conditions, and perform the unconfined compression simulation. The elastin fiber network of the 38-year-old sample before and after compression is shown in Fig. 4(b) and (c), respectively. This simulation setup facilitated a detailed analysis of the contribution of elastin fibers to the skin’s mechanical properties under compression.

### Quantification of elastin network architecture

Following the reconstruction of the elastin fiber network, several quantitative metrics were calculated for each of the nine samples to characterize their 3D architecture. These parameters are defined as follows:

- Mean Fiber Diameter: The diameter of each individual fiber was measured locally from the segmented 3D image data. This resulted in a distribution of diameters within each sample. For the purpose of comparison between donors, the mean fiber diameter for the entire network was calculated for each sample.
- Fiber Count: The total number of individual beam elements representing elastin fibers within the model.
- Elastin Volume Fraction: The total volume of all elastin fibers divided by the total volume of the dermal matrix model, expressed as a percentage.
- Fiber Clusters and Connectivity: The final reconstructed network of beam elements was treated as a 3D graph to analyze its connectivity. In this framework, a “cluster” is defined as a connected component–a subset of fibers in which a continuous path exists from any fiber to any other fiber within that same subset. The number of clusters, a measure of network fragmentation, was identified computationally using a standard Breadth-First Search (BFS) algorithm^36^.
- Maximum Cluster Size: The number of fibers contained within the single largest connected component (cluster).
- Vertical Fiber Proportion: The orientation of each fiber was calculated relative to the normal of the skin surface (the Y-axis in the model). Fibers with an orientation angle between $0^\circ$ and $45^\circ$ relative to the surface normal were classified as “vertical.” The vertical fiber proportion is the percentage of total fibers that meet this criterion. While more comprehensive metrics for anisotropy exist (e.g., orientation tensors), this specific metric was chosen for its direct mechanical relevance to the unconfined vertical compression simulation performed in this study, as it provides a clear measure of the network’s ability to resist deformation in the primary loading direction.

It is important to note that these connectivity-based metrics are inherently sensitive to the parameters used during the upstream image processing and segmentation steps. The choice of image threshold and the application of morphological operations (e.g., closing) directly influence whether small gaps between fibers are bridged, thereby affecting the final number and size of the calculated clusters. Therefore, the consistent and standardized application of our image processing pipeline across all nine samples was critical to ensure a valid comparison of network architecture.

## Results

### Age-related changes in elastin fiber characteristics

Figure 3 indicates that younger skin models (e.g., 38–49 years) exhibit a denser and more interconnected elastin fiber network, with numerous vertically oriented fibers contributing to structural integrity. As age increases, the number of elastin fibers gradually decreases, and their connectivity becomes less structured and more fragmented. In the older skin models (e.g., 67–78 years), the fiber network appears sparser and more disorganized, with a noticeable reduction in fiber density and length, particularly in the upper dermal region. Moreover, the fiber orientation shifts, with an increasing prevalence of horizontally aligned fibers in older skin models (e.g., 67–78 years), indicating a loss of structural support in the vertical direction. The quantitative data of elastin fibers in the skin across different ages, including averaged fiber diameter, fiber count, volume fraction, clustering characteristics, and the proportion of vertically oriented fibers are provided in Table 1. The vertically oriented fibers were defined by an angle threshold ($45^{\circ }$) relative to the skin surface normal.

**Table 1:** Summary of age-dependent elastin fiber microstructural and mechanical properties. The table presents quantitative metrics of elastin fiber networks across different age groups, including average fiber diameter, total fiber count, elastin volume fraction, number of fiber clusters, maximum cluster size (number of fibers in the largest connected component), and the proportion of vertically oriented fibers.

Age (years)	Mean Fiber Diameter ($\mu$m)	Fiber Count	Elastin Volume Fraction (%)	Number of Fiber Clusters	Maximum Cluster Size	Vertical Fiber Proportion (%)	Normalized Skin Firmness
38	3.86	2437	4.23	5	2415	25.44	1.0
39	3.54	1060	2.34	17	795	28.30	0.155
43	3.68	2183	5.26	11	2127	18.51	0.837
49	3.59	2056	3.26	28	1642	32.48	0.533
58	3.45	2081	4.18	32	1883	26.86	0.301
59	3.52	1713	3.63	19	1070	16.40	0.318
67	3.35	804	2.28	47	248	6.22	0.0256
70	3.37	1352	2.25	40	626	17.31	0.044
78	3.36	1070	2.32	51	303	9.63	0.0

The data demonstrate an age-related decline in elastin fiber integrity, characterized by reductions in fiber diameter, fiber count, and elastin volume fraction, all of which contribute to diminished skin skin firmness. The mean fiber diameter decreases from 3.86 $\mu m$ at 38 years to 3.35 $\mu m$ at 67 years, suggesting a thinning of elastin fibers with aging. The full range (minimum and maximum) of the measured diameters for each donor is provided in the Supplementary Information (Supplementary Table S3). Similarly, the number of fibers decreases significantly, from 2437 at 38 years to 1070 at 78 years, indicating elastin degradation. The fiber volume fraction follows a similar trend, with the older group exhibiting approximately half the volume fraction observed in the younger group. This observation is consistent with the progressive decline in elastin content. Additionally, the number of fiber clusters increases dramatically with age, from 5 clusters at 38 years to 51 clusters at 78 years, reflecting increased fragmentation of the elastin network. The maximum number of fibers within a single cluster also declines sharply with age, suggesting progressive breakdown of elastin fiber connectivity that supports skin firmness. Notably, the proportion of vertical fibers, which are essential for resisting mechanical deformation and gravitational forces, declines to less than 10.0% at 78 years. This reduction in vertical fiber content contributes to skin sagging and loss of resilience in aging individuals. In this study, the mechanical performance of each skin model was evaluated using a dimensionless metric referred to as normalized skin firmness. The normalized skin firmness was defined based on the maximum and minimum elastic recovery forces observed among all samples, where a value of 1.0 represents the highest recorded skin firmness (corresponding to the model with the greatest resistance to compression), and 0 denotes the lowest skin firmness (indicating the weakest resistance). The elastic recovery force for each sample was computed as the total reaction force acting on the top surface of the FE model during simulated unconfined compression. The computed skin firmness showed a clear decreasing trend with increasing age. This pattern is consistent with the known fact that skin firmness typically declines with age, which is considered to be associated with the properties of elastin fibers. To investigate whether fiber diameter correlates with orientation, an additional analysis was performed on the representative 38-year-old sample. Fibers were categorized as ’vertical’ (orientation angle < $45^\circ$ relative to the skin normal) or ’horizontal’ (angle >= $45^\circ$). An independent samples t-test was subsequently used to compare the mean diameters of these two fiber populations. The analysis revealed no statistically significant difference between the mean diameter of vertical and horizontal fibers (p > 0.05), suggesting that fiber thickness is independent of its orientation within the network. To further investigate the presence of any preferential fiber alignment in the plane parallel to the skin surface, the orientation of fibers in the horizontal (X-Z) plane was analyzed for all nine samples (see Supplementary Figure S1). The orientation angle was calculated for the projection of each fiber vector onto this plane. The analysis revealed that the networks were generally isotropic in the horizontal plane. However, a mild to moderate degree of anisotropy was observed in several samples (ages 49, 58, and 78), which showed a preferential alignment of fibers along the $90^\circ$ axis. This suggests that while a random orientation is common, some structural organization can exist in the horizontal plane.

**Fig. 5:**
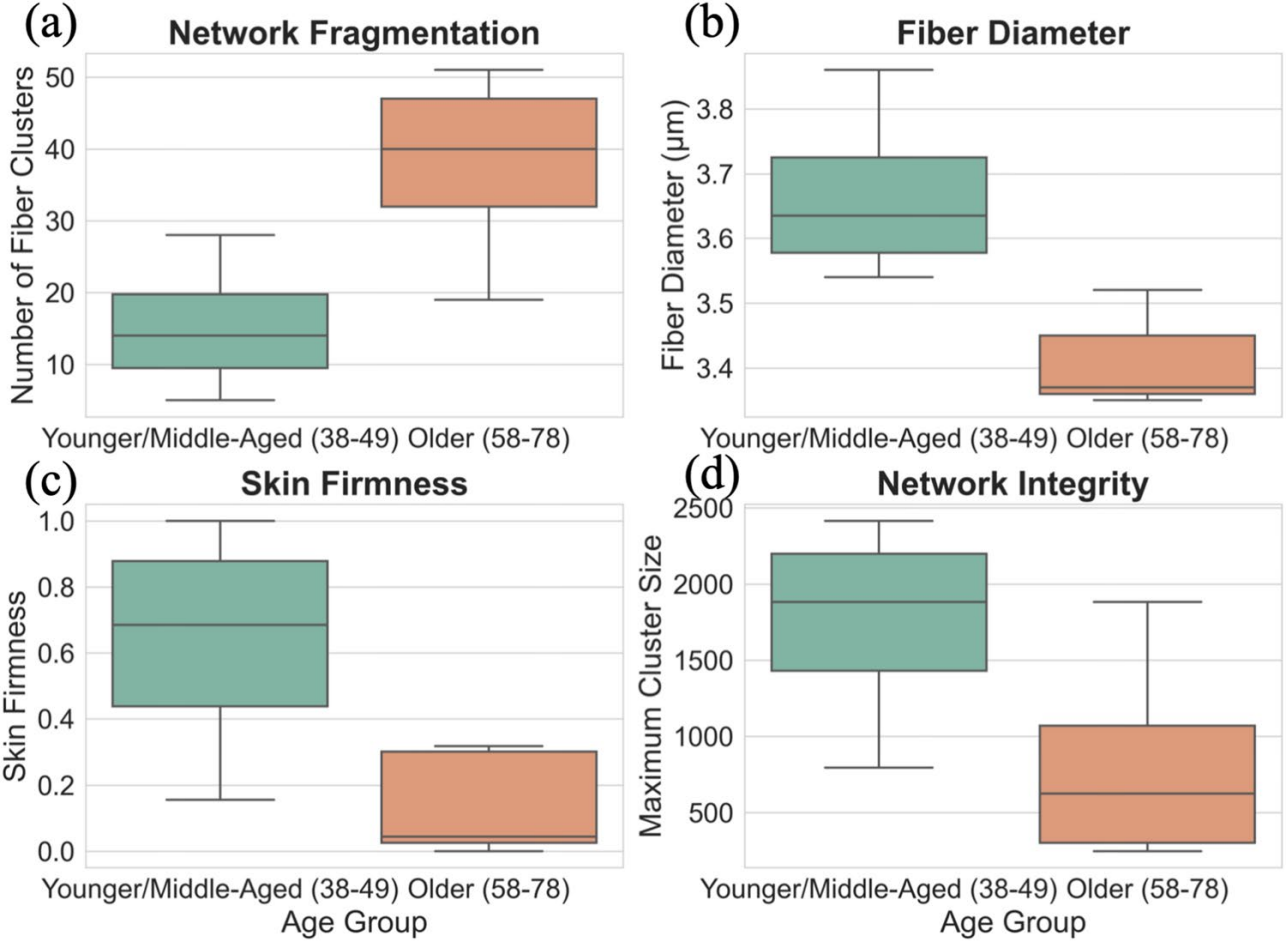
Comparison of key parameters between age groups. Boxplots showing the distribution of (**a**) number of fiber clusters, (**b**) fiber diameter, (**c**) normalized skin firmness, and (**d**) maximum cluster size for the Younger/Middle-Aged (38-49 years; n=4) and Older (58-78 years; n=5) cohorts. The boxes represent the interquartile range (IQR), the horizontal line is the median, and the whiskers extend to 1.5 times the IQR. The results show a significant increase in fragmentation (**a**) and decrease in fiber diameter (**b**) in the older cohort, with corresponding trends of declining skin firmness (**c**) and network integrity (**d**).

To further investigate the age-related changes, samples were categorized into a ’Younger/Middle-Aged’ group (n=4, ages 38-49) and an ’Older’ group (n=5, ages 58-78). The means of key structural and mechanical parameters were then compared between these two groups using independent samples t-tests. The analysis revealed a statistically significant increase in network fragmentation in the older cohort, as measured by the number of fiber clusters (Younger/Middle-Aged mean = 15.25 ± 9.81; Older mean = 37.80 ± 12.76; p = 0.020). Furthermore, the fibers themselves were significantly thinner in the older group, with a lower average fiber diameter (Younger/Middle-Aged mean = 3.67 ± 0.14 $\upmu$m; Older mean = 3.41 ± 0.07 $\upmu$m; p = 0.027). While not reaching the threshold for statistical significance, strong trends were observed for other key metrics. The skin firmness showed a clear decline in the older group (p = 0.070), and the mean maximum cluster size, a measure of network integrity, was substantially smaller in the older cohort (p = 0.093). The mean fiber count was also lower in the older group, though this difference was not statistically significant (p = 0.211), likely due to the high inter-individual variability observed within the small sample size. To visually compare the characteristics of the elastin network between the two age cohorts, boxplots were generated for key structural and mechanical parameters (Fig. 5). These plots visually confirm the results of the statistical tests, illustrating a significant increase in network fragmentation in the Older cohort, as shown by the higher number of fiber clusters (Fig. 5a), and a significant decrease in fiber diameter (Fig. 5b). Furthermore, the boxplots clearly show a trend towards lower skin firmness (5c) and a substantially reduced maximum cluster size (Fig. 5d) in the Older group compared to the Younger/Middle-Aged group. These plots effectively highlight the differences in both the central tendency and the data spread for each group, supporting the conclusion that the structural and mechanical integrity of the elastin network is significantly compromised in the older cohort. Overall, this group-based analysis provides statistical evidence supporting the observation that skin from the older cohort exhibits significantly increased fiber fragmentation and reduced fiber thickness, which corresponds to the observed decline in skin firmness.

### Mechanical effect of elastin fiber on the skin deformation

**Fig. 6:**
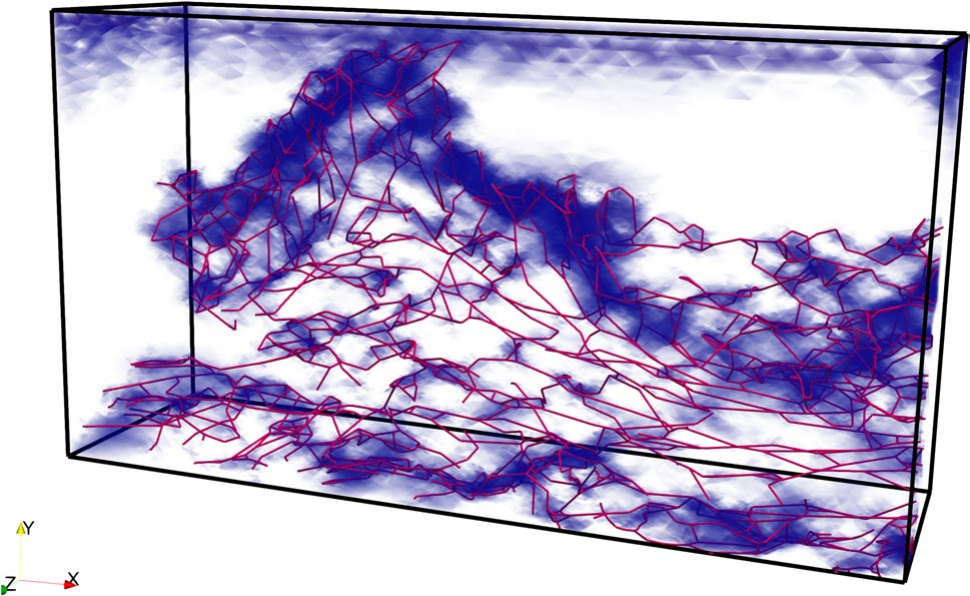
Spatial correlation between elastin fiber architecture and low-strain regions under compressive loading. The red lines denote the centerlines of elastin fibers reconstructed from 3D imaging data, while the blue shaded areas represent regions of low strain (equivalent strain magnitude < 0.1) identified from FE simulation results. The figure illustrates how densely packed and interconnected fiber networks correspond to zones of reduced deformation, highlighting the mechanical shielding effect of elastin fibers in maintaining dermal integrity under compressive stress.

Figure 6 demonstrates the spatial correlation between elastin fiber structure and the distribution of low strain regions within the dermis of the 38-year-old subject. The red lines represent the centerlines of the reconstructed elastin fibers, while the blue regions correspond to low-strain areas, defined by an equivalent Von Mises strain magnitude below a threshold of 0.1. The overlay reveals a strong spatial correlation between the location of elastin fibers and the emergence of low-strain zones. Specifically, the dense clustering and branching of fibers, particularly in the upper-central and basal regions of the dermis, coincide with extended low-strain areas. This suggests that the elastin network plays a crucial role in locally modulating mechanical deformation, acting as a reinforcing structure that redistributes and limits the strain in its immediate vicinity. On the other hand, the areas with fewer or no fibers show higher strain levels. These findings emphasize the importance of fiber presence and orientation in maintaining structural integrity and resisting deformation.

**Fig. 7:**
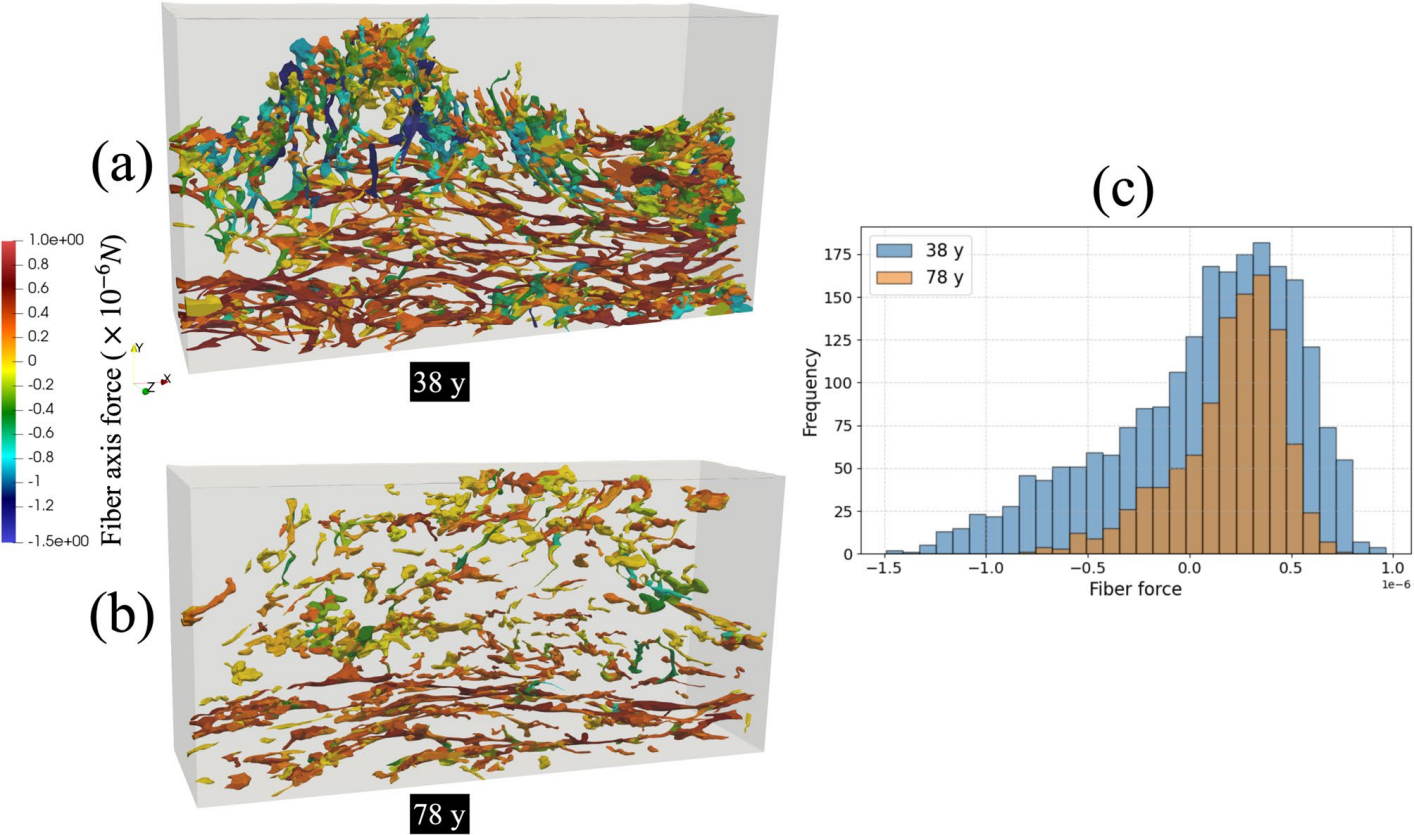
Comparison of fiber force distributions in elastin networks reconstructed from skin samples of a 38-year-old (**a**) and a 78-year-old (**b**) subject. The visualizations display the mesh elements representing elastin fibers, color-mapped according to the computed fiber force values from beam elements, ranging from high compression (blue) to high tension (red). The younger network in (**a**) shows a more extensive and interconnected force distribution than the aged network in (**b**). Histogram comparing the distribution of axial forces for all fibers in the 38-year-old (blue) and 78-year-old (orange) samples is displayed in panel (**c**). The broader distribution for the 38-year-old sample, particularly in the compressive region (negative forces), provides a quantitative confirmation that the younger, more intact network engages more fibers in significant load-bearing compared to the aged, fragmented network.

The comparison of fiber force distributions between the 38-year-old and 78-year-old samples highlights distinct differences in the spatial patterns and localization of tensile and compressive forces within the elastin network (Fig. 7). In the younger sample (38 y), compressive forces–represented by cooler colors in Fig. 7 predominantly concentrated in the upper and central regions of the dermis, corresponding to areas of dense fiber interconnectivity and vertical fiber bundles. These regions experience compressive stress due to the resistance against vertical deformation under external loading from the skin surface. Tensile forces indicated by warmer colors distributed more broadly along the horizontal, basal regions of the network. In contrast, the aged sample (78 y) exhibits a fragmented and spatially discontinuous force distribution. Tensile forces appear weaker and more sparsely distributed than in the younger skin. Compressive forces are minimal and limited to isolated clusters, reflecting the lack of vertical fibers and inter-fiber interactions. This difference is quantified in the force distribution histogram (Fig. 7c). The 38-year-old sample displays a broad distribution of forces, with a substantial population of fibers experiencing significant compressive loads. This indicates that a large portion of the network is actively resisting the applied deformation. The 78-year-old sample, however, shows a much narrower distribution with far fewer fibers in a state of high compression. This quantitatively confirms that the older, more fragmented network has a diminished capacity to bear compressive loads, consistent with the observed loss of vertical fibers and overall structural integrity.

These fiber force distribution results also indicate distinct mechanical responses in the fibers based on their orientation (Fig. 7). The horizontally oriented fibers appear in red, signifying that they are under tensile stress and being stretched. This is because when the sample is compressed in the longitudinal direction under incompressible conditions, it extends in the transverse direction. In contrast, the vertically oriented fibers are displayed in blue, indicating that they are experiencing compressive forces and being compressed due to the vertical deformation. This suggests a directional load transfer within the fiber network, where horizontal fibers bear the stretching forces while vertical fibers resist compression. These findings provide insight into the mechanical interactions of fibers under deformation and highlight the role of fiber orientation in structural stability and load distribution.

### Relationship between the elastin fibers’ characteristics and the skins’ firmness

**Fig. 8:**
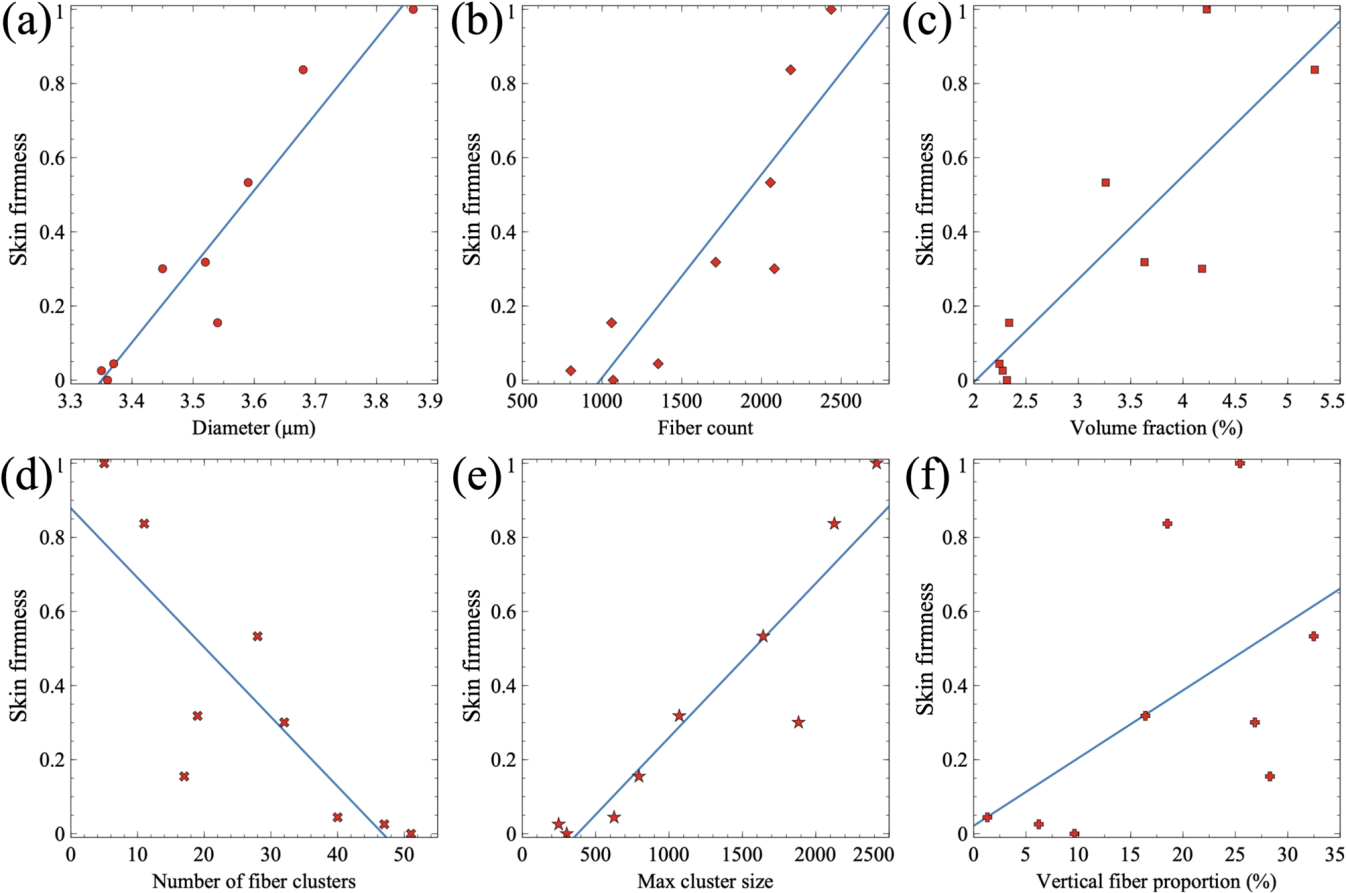
Correlation between skin firmness and various elastin fiber parameters. (**a**) Skin firmness versus fiber diameter, showing a positive correlation. (**b**) Skin firmness versus fiber count, indicating that a higher number of fibers enhances mechanical properties. (**c**) Skin firmness versus fiber volume fraction, demonstrating that a greater elastin content contributes to increased firmness. (**d**) Firmness versus the number of fiber clusters, revealing a negative correlation, suggesting that fragmentation weakens the elastin network. (**e**) Skin firmness versus maximum cluster size, indicating that larger, interconnected fiber networks improve mechanical support. (**f**) Skin firmness versus the proportion of vertical fibers, showing that a higher percentage of vertically aligned fibers enhances resistance to compressive forces.

Figure 8 illustrates the relationship between skin firmness and various elastin fiber parameters, revealing distinct trends. A strong positive correlation is observed between fiber diameter and skin firmness, indicating that thicker elastin fibers contribute to higher mechanical resilience (Fig. 8a). Similarly, the fiber count exhibits a direct correlation with skin firmness, suggesting that a greater number of elastin fibers enhances the skin firmness (Fig. 8b). The fiber volume fraction also follows this trend, where a higher proportion of elastin fibers within the dermis is associated with increased skin firmness (Fig. 8c). In contrast, Fig. 8(d) shows an inverse relationship between skin firmness and the number of fiber clusters, suggesting that fragmentation of the elastin network reduces its ability to sustain mechanical loads. On the other hand, the maximum cluster size (number of fibers in the largest connected component) is positively correlated with skin firmness, indicating that larger, more interconnected fiber networks provide superior mechanical support (Fig. 8e). Lastly, Fig. 8(f) demonstrates a positive correlation between the proportion of vertically oriented fibers and skin firmness, highlighting the crucial role of fiber alignment in maintaining skin’s resistance to compressive forces. These results collectively suggest that the deterioration of elastin fiber structure with age, characterized by a reduction in fiber diameter, count, and connectivity, is a key contributor to the decline in skin firmness.

To quantify these trends, a Pearson correlation analysis was performed to evaluate the relationship between the quantified elastin fiber parameters and the normalized firmness values from the FE simulations (Table 2). Pearson correlation coefficients ($r=\frac{\sum \left( x_{i}-\bar{x}\right) \left( y_{i}-\bar{y}\right) }{\sqrt{\sum \left( x_{i}-\bar{x}\right) ^{2} \sum \left( y_{i}-\bar{y}\right) ^{2}}}$, $x_{i}, y_{i}$ are individual data points and $\bar{x}, \bar{y}$ are means of *x* and *y* respectively) were calculated to determine the strength and direction of the relationships. The p-value was calculated for each correlation to test for statistical significance. The analysis reveals statistically significant correlations for all tested parameters since all p-values are less than 0.05.

To further investigate the effect of each fiber parameter on the skin firmness, a simple linear regression was performed. This analysis was used to generate the best-fit trend lines visually depicted in Fig. 8. The regression slopes ($\beta$) were also derived from this analysis to compare the relative influence of different structural parameters on skin firmness. This analysis further clarified the relative importance of these factors, identifying fiber count (the slope of the regression line $\beta =0.48$) and maximum cluster size ($\beta =0.41$) as the strongest predictors of skin firmness. This underscores their synergistic contribution: fiber count establishes the fundamental density of the network, while maximum cluster size reflects its structural continuity and resistance to fragmentation-induced failure. While fiber orientation, specifically the vertical proportion of fibers, is mechanistically important for counteracting specific forces, its influence was found to be secondary to that of overall network density and integrity. This suggests that the primary driver of skin firmness loss observed here is the age-related decrease in fiber number coupled with increased fragmentation, which erodes the network’s core structural cohesion.

**Table 2:** Statistical analysis of correlations between elastin fiber parameters and skin firmness.

Parameter vs. Skin Firmness	Pearson’s r	p-value
Fiber Diameter	0.84	< 0.01
Fiber Count	0.94	< 0.001
Elastin Volume Fraction	0.81	< 0.01
Number of Fiber Clusters	-0.92	< 0.001
Maximum Cluster Size	0.95	< 0.001
Vertical Fiber Proportion	0.82	< 0.01

## Discussion

### Age-induced deterioration of the elastin network and its implications

Our results also demonstrate a strong correlation between aging and the deterioration of elastin fibers, consistent with previous dermatological studies^37,38^. The results (Table 1) reveal a clear age-related decline in elastin fiber integrity, characterized by reductions in fiber diameter, total fiber count, and elastin volume fraction. As age increases, fibers become more fragmented, with a rising number of isolated fiber clusters and a decrease in the maximum cluster size, indicating structural disorganization. Notably, the proportion of vertically oriented fibers decreases with age, which also contributes to diminished skin firmness and mechanical resistance during compression. Therefore the decrease of vertically oriented fibers results in an increase of skin laxity. Overall, the reduction in fiber diameter, count, and volume fraction directly contributes to the loss of skin firmness^39^. Aging processes such as enzymatic degradation, oxidative stress, glycation, and mechanical fatigue contribute to the deterioration of elastin fibers. These degradative processes lead to fragmentation and disorganization of the elastin network, compromising the skin’s mechanical properties^40^. The observed trends align with previous studies on skin aging, reinforcing the notion that elastin degradation is a primary factor in age-related loss of skin firmness^41,42^. These findings suggest that targeted interventions aimed at preserving elastin fiber structure could be crucial for maintaining the skin firmness.

While our results demonstrate a clear overall trend of declining elastin network integrity with age, it is crucial to acknowledge the significant inter-individual variability present in the data. This is particularly evident in the sample from the 39-year-old donor (Fig. 3b), which presents with a fiber count (1060) and elastin volume fraction (2.34%) more characteristic of an individual in the 60s or 70s. Such variations are expected in human studies and can be attributed to a combination of intrinsic factors (e.g., genetic predisposition) and extrinsic lifestyle factors. Cumulative sun exposure (photoaging), for example, is a well-established accelerator of enzymatic degradation and fragmentation of elastin fibers.

### Elastin’s architectural influence on skin firmness

The relationship between the microstructural architecture of dermal elastin fibers and the macroscopic mechanical property of skin firmness is a critical aspect of skin physiology and aging. Our analysis provides quantitative insights into this relationship, revealing how specific characteristics of the elastin network contribute to or detract from skin resilience.

Consistent with fundamental biomechanical principles, we observed significant positive correlations between skin firmness and elastin fiber diameter (Fig. 8a), fiber count (Fig. 8b), and overall elastin volume fraction (Fig. 8c). These findings suggest that a greater abundance of thicker elastin fibers forms a denser, more robust network within the dermal extracellular matrix (ECM). Such a network possesses enhanced load-bearing capacity and is better equipped to resist deformation and store elastic energy, efficiently returning the skin to its original state after stretching. This aligns with established knowledge regarding the function of cross-linked elastin in providing recoil and dissipating mechanical stress within connective tissues^43,44^. Conversely, a key indicator of network degradation, the number of distinct fiber clusters, exhibited a strong negative correlation with skin firmness (Fig. 8d). An increase in cluster number signifies greater fragmentation of the elastin network. This fragmentation disrupts the continuity of load-bearing pathways, potentially reducing the network’s ability to effectively distribute mechanical forces. Such structural breakdown is a known hallmark of both chronological aging and photoaging, often mediated by increased activity of MMPs like elastase and potentially exacerbated by factors like glycation and oxidative stress^45–48^. This compromised connectivity directly reduces resilience. Further supporting the importance of network integrity, the maximum size of connected fiber clusters demonstrated a strong positive correlation with skin firmness (Fig. 8e; Pearson’s $r=0.95$). This indicates that even in the presence of some fragmentation (increased cluster number), the existence of large, contiguous elastin aggregates helps maintain mechanical function. These larger clusters serve as essential structural hubs, preserving significant load-bearing and recoil capabilities within the tissue. Beyond density and continuity, fiber organization also plays a significant role. The proportion of vertically oriented elastin fibers showed a positive correlation with skin firmness (Fig. 8f; Pearson’s $r=0.82$). This observation highlights the anisotropic nature of the dermal elastin network and suggests a specific functional adaptation. Vertically aligned fibers are biomechanically positioned to counteract gravitational pull and resist compressive forces, contributing significantly to maintaining skin firmness and preventing sagging, particularly perhaps at critical locations like the dermal-epidermal junction.

Integrating these findings, our quantitative analysis highlights the critical interdependence between multiple architectural features of the elastin network and skin’s elastic performance. The stark contrast between the subjects at the extremes of the age range studied exemplifies this relationship: the 38-year-old subject exhibited the highest normalized firmness, corresponding to an optimal elastin architecture characterized by the maximal fiber count (2437 fibers), the largest intact cluster size (2415 fibers, indicating minimal fragmentation), and a substantial proportion of vertically oriented fibers (25.44%). This represents a dense, highly integrated, and appropriately organized network capable of efficiently handling mechanical stress. In contrast, the 78-year-old subject, with the lowest firmness, displayed severely compromised architecture: a dramatically reduced fiber count (1070 fibers), extensive fragmentation (indicated by 51 distinct clusters), and a diminished proportion of vertical fibers (9.63%). These observations are congruent with histopathological models of skin aging, which describe a decline in elastin synthesis alongside accelerated enzymatic degradation, leading to a simultaneous loss of both the quantity and the topological integrity of elastin fibers^16,49^.

Collectively, these findings emphasize that maintaining skin firmness is contingent not just on the amount of elastin present, but critically on its architectural organization and integrity. Age-related decline in skin mechanical properties appears strongly linked to the progressive fragmentation and disorganization of the elastin fibers. This mechanistic understanding provides a strong rationale for therapeutic and cosmetic strategies aimed at counteracting skin aging by preserving or restoring elastin network integrity. Such approaches could include stimulating neosynthesis of elastin (e.g., via growth factors or retinoids^50^), inhibiting proteolytic degradation (e.g., through MMPs inhibitors^51–53^), preventing damage (e.g., robust photoprotection, antioxidants^54^), or potentially employing biomimetic scaffolds or tissue engineering techniques^55,56^ to restore a functionally anisotropic fiber network.

It is important to acknowledge the limitations of our study to properly contextualize the findings. Our analysis was conducted on a specific and limited cohort: nine Caucasian female donors aged 38 to 78 years. The absence of data from younger donors, particularly those in their 20s and 30s, means our study captures the progression of elastin degradation in middle to older age but lacks a baseline representing peak dermal integrity. The process of elastin degradation is known to worsen after 40 years of age, and a younger cohort would provide critical insight into the initial stages of this decline. It is also important to consider the source of the tissue and the potential influence of confounding donor characteristics on our findings. The BMI of the donors ranged from 26 to 35, indicating the cohort was composed of overweight or obese individuals. As BMI can influence skin properties, we performed a correlation analysis between the donors’ BMI and the measured elastin network parameters and normalized skin firmness (see Supplementary Table S4 for full results). This analysis did not reveal any statistically significant correlations within our cohort (p> 0.05 for all tests), suggesting that within this specific BMI range, BMI was not a primary driver of the observed variations. However, we acknowledge other limitations regarding donor history. Information on the donors’ smoking status and any use of hormone therapy–both of which are known to affect skin aging–was not available. The absence of this data prevents us from controlling for these potential confounding variables. Therefore, while our results show a strong correlation with chronological age, the influence of these un-tracked lifestyle and physiological factors cannot be fully excluded and should be addressed in future studies.

## Conclusion

This study investigated the relationship between age-related changes in the geometric architecture of dermal elastin fibers and skin firmness using a computational approach that integrated realistic fiber geometries obtained from 3D imaging data into FE models. Human abdominal skin samples from women aged 38 to 78 years were analyzed. Unconfined compression simulations were performed to assess skin firmness, which was correlated with quantified microstructural parameters of the elastin network. The main findings of the study are as follows:

- Quantitative analysis revealed a significant decline in elastin network integrity with age, characterized by decreased average fiber diameter, reduced total fiber count, and lower elastin volume fraction. Furthermore, aging was associated with increased fragmentation of the network (higher number of fiber clusters, smaller maximum cluster size) and a decrease in the proportion of vertically oriented fibers.
- Skin firmness, evaluated via simulated compression tests, showed a clear decline with age. Strong positive correlations were found between normalized firmness and elastin fiber diameter, fiber count, volume fraction, maximum cluster size, and the proportion of vertical fibers. Conversely, skin firmness was negatively correlated with the number of fiber clusters, indicating that network fragmentation compromises mechanical resilience.
- FE analysis demonstrated the mechanical role of elastin fibers in modulating strain distribution, with denser fiber networks correlating with low-strain regions. Fibers’ force analysis highlighted that horizontal fibers primarily bear tensile loads, while vertical fibers experience compressive force under the unconfined compression condition. Regression analysis identified fiber count and maximum cluster size as the strongest predictors of firmness, emphasizing the importance of fiber network density and continuity.

Our results demonstrate that maintaining skin firmness is contingent not only on the quantity of elastin but critically on its microstructural organization. By integrating realistic fiber geometries, this study provides a quantitative, mechanistic understanding linking specific geometric features of the elastin network to macroscopic skin mechanics, offering a rationale for developing therapeutic and cosmetic strategies aimed at preserving or restoring elastin network integrity to counteract skin aging. Several limitations of this study should be acknowledged. First, our model focuses exclusively on the explicit geometry of the elastin network within a homogeneous dermal matrix. However, in vivo aging involves complex and simultaneous alterations to all components of the extracellular matrix–including the collagen network (e.g., increased cross-linking, glycation, and disorganization) and the ground substance. Our current model does not incorporate the degradation or remodeling of the collagen network. Second, the dermal matrix was modelled using the Saint Venant–Kirchhoff constitutive formulation, which assumes infinitesimal strain and may be insufficient to capture the nonlinear and time-dependent behavior of the dermal ground substance. Employing a more advanced viscoelastic or hyperelastic material model could improve fidelity under larger deformations. Nevertheless, our computational framework was intentionally designed to isolate a single variable: the effect of age-related changes in the three-dimensional architecture of the elastin network. By holding the properties of the surrounding matrix constant across all simulations, we were able to test the hypothesis that geometric degradation of elastin fibers alone can significantly impair the skin’s firmness.

## Supplementary Information

Supplementary Information.

## Data Availability

The datasets used and/or analyzed during the current study are available from the corresponding author on reasonable request.
