# Supplementary material for: Influence of aging on dermal elastin fiber architecture and skin firmness assessed by finite element modeling
Source: Sci Rep. 2025 Aug 5;15:28598. doi: 10.1038/s41598-025-14393-2 (PMC12325628; doi:10.1038/s41598-025-14393-2)
Supplement: Supplementary file 1 — Supplementary Information. [file 41598_2025_14393_MOESM1_ESM.pdf]

Supplementary information

Table S1. Total number of elements for each of the nine finite element models.

Age (years)	38	39	43	49	58	59	67	70	78
Total number of elements	1,200,459	1,099,533	909,887	1,204,645	1,207,685	1,062,440	929,613	930,440	1,012,309

Table S2. The height of the computational domain for each donor sample, determined by the depth of the 3D confocal image acquisition.

Age (years)	38	39	43	49	58	59	67	70	78
Height (µm)	268.32	238.22	201.67	268.32	236.07	261.87	264.02	272.62	246.82

Table S3. The range (minimum and maximum) of measured fiber diameters for each donor sample.

Age (years)	Minimum Diameter (μm)	Maximum Diameter (μm)
38	2.38	4.29
39	2.02	3.48
43	2.65	4.57
49	2.24	3.85
58	2.38	4.10
59	2.52	4.34
67	2.17	3.74
70	2.23	3.85
78	1.76	3.03

Table S4. Correlation analysis between donor Body Mass Index (BMI) and measured skin parameters. No statistically significant correlations were found ($p > 0.05$ for all tests).

Parameter	Pearson's r	p-value
Fiber Diameter	0.042	0.914
Fiber Count	0.024	0.950
Elastin Volume Fraction	-0.025	0.949
Number of Fiber Clusters	0.095	0.807
Maximum Cluster Size	-0.028	0.942
Vertical Fiber Proportion	0.147	0.706
Normalized Firmness	0.021	0.957

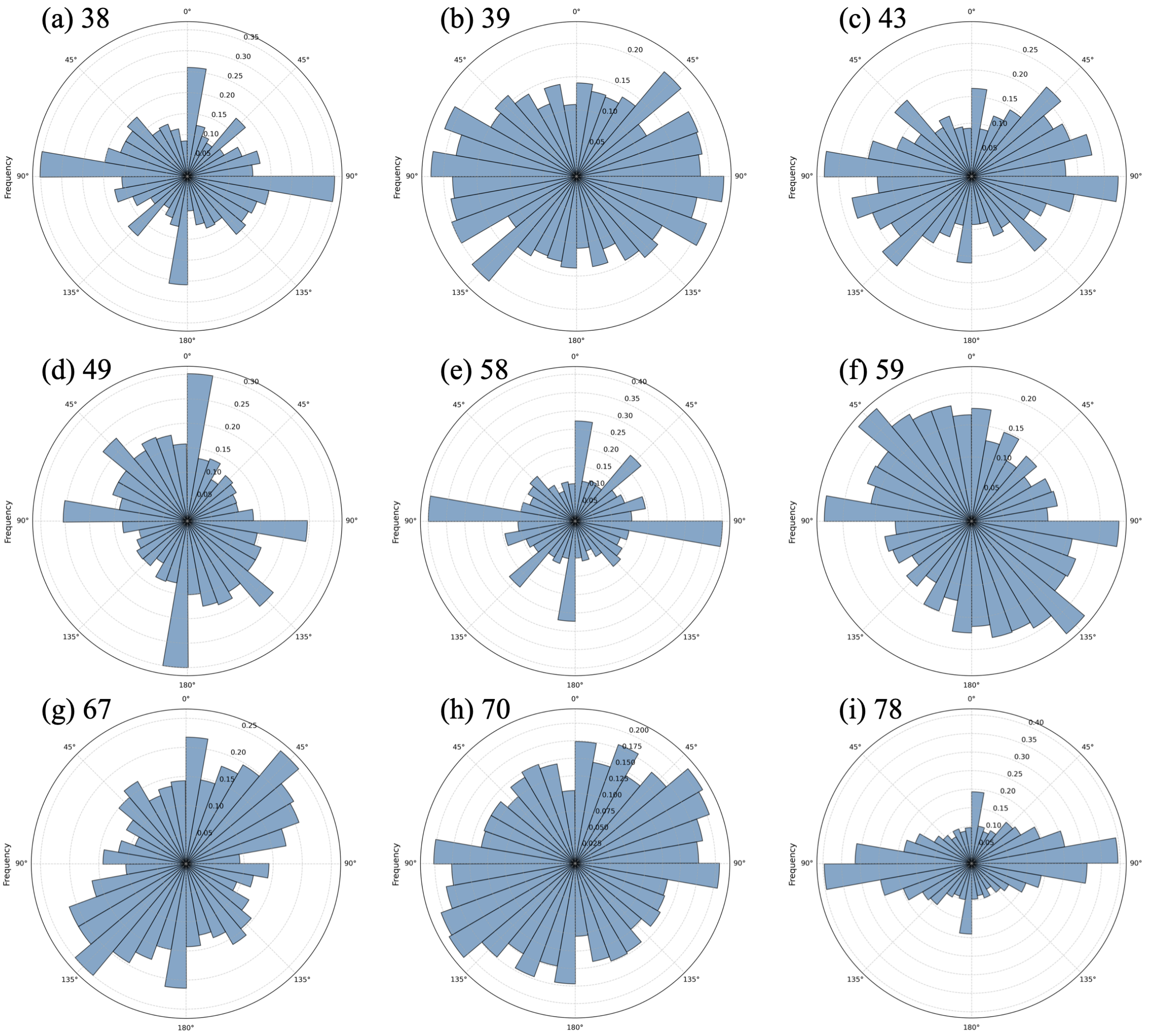

Figure S1. Rose plots of the elastin fiber orientation in the horizontal (X-Z) plane for all nine donor samples. The orientation angle was calculated for the projection of each fiber vector onto this plane. The radial length of each wedge represents the frequency of fibers oriented in that direction. While many samples show a generally uniform (isotropic) distribution, several cases (e.g., (d), (e), (i)) exhibit a mild degree of anisotropy with a preferential alignment along the 90° axis. This suggests that while a random orientation is common, some structural organization can exist in the horizontal plane.
